# 3α,4α-Ep­oxy-5α-androstan-17β-yl acetate

**DOI:** 10.1107/S160053680900899X

**Published:** 2009-03-25

**Authors:** L. C.R. Andrade, J. A. Paixão, M.J.M. de Almeida, F. M. Fernandes Roleira, E. J. Tavares da Silva

**Affiliations:** a CEMDRX, Departamento de Física, Faculdade de Ciências e Tecnologia, Universidade de Coimbra, P-3004-516 Coimbra, Portugal; b Centro de Estudos Farmacêuticos, Laboratório de Química Farmacêutica, Faculdade de Farmácia, Universidade de Coimbra, P-3000-295 Coimbra, Portugal

## Abstract

The title compound, C_21_H_32_O_3_, results from modifications of the *A* and *D* rings of the aromatase substrate androstenedione. Ring *A* adopts a conformation between 10β-sofa and 1α,10β half-chair. Rings *B* and *C* are in slightly flattened chair conformations. Ring *D* approaches a 13β-envelope conformation, probably due to the acet­oxy substituent, and shows a very short C*sp* ^3^—C*sp* ^3^ bond next to the epoxide ring, which is characteristic of 3–4 epoxides. .

## Related literature

For the antitumor and anti-aromatase activity of aromatase substrate derivatives, see: Cepa *et al.* (2005 ▶). For related structures, see: Paixão *et al.* (1997 ▶); Andrade *et al.* (1997 ▶). For bond-length data,, see: Allen *et al.* (1987 ▶). For asymmetry, pseudo-rotation and puckering parameters, see: Duax & Norton (1975 ▶); Cremer & Pople (1975 ▶); Altona *et al.* (1968 ▶).
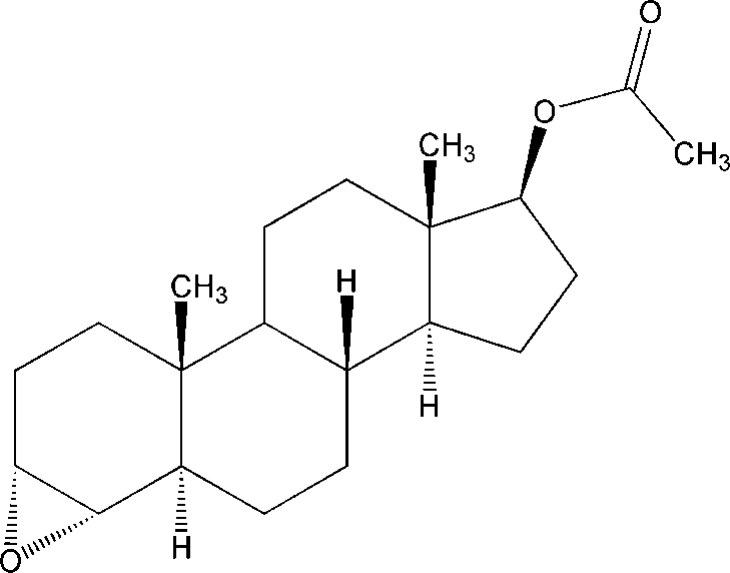


## Experimental

### 

#### Crystal data

C_21_H_32_O_3_
*M* *_r_* = 332.47
Orthorhombic, 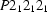

*a* = 6.2760 (2) Å
*b* = 11.7272 (19) Å
*c* = 25.0888 (9) Å
*V* = 1846.5 (3) Å^3^
*Z* = 4
Cu *K*α radiation
μ = 0.61 mm^−1^
*T* = 293 K
0.36 × 0.20 × 0.12 mm

#### Data collection

Enraf–Nonius MACH-3 diffractometer
Absorption correction: none
2661 measured reflections
2146 independent reflections
1715 reflections with *I* > 2σ(*I*)
*R*_int_ = 0.050
3 standard reflections every 300 reflections intensity decay: 1.3%

#### Refinement

*R*[*F* ^2^ > 2σ(*F* ^2^)] = 0.044
*wR*(*F* ^2^) = 0.135
*S* = 1.03
2146 reflections
221 parameters
H-atom parameters constrained
Δρ_max_ = 0.23 e Å^−3^
Δρ_min_ = −0.18 e Å^−3^
Absolute structure: Flack (1983 ▶), with 0 Friedel pairs
Flack parameter: −0.1 (5)

### 

Data collection: *CAD-4 Software* (Enraf–Nonius, 1989 ▶); cell refinement: *CAD-4 Software*; data reduction: *HELENA* (Spek, 1997 ▶) and *PLATON* Spek (2009 ▶); program(s) used to solve structure: *SHELXS97* (Sheldrick, 2008 ▶); program(s) used to refine structure: *SHELXL97* (Sheldrick, 2008 ▶); molecular graphics: *ORTEPII* (Johnson, 1976 ▶); software used to prepare material for publication: *SHELXL97*.

## Supplementary Material

Crystal structure: contains datablocks I, global. DOI: 10.1107/S160053680900899X/kp2201sup1.cif

Structure factors: contains datablocks I. DOI: 10.1107/S160053680900899X/kp2201Isup2.hkl

Additional supplementary materials:  crystallographic information; 3D view; checkCIF report

## Figures and Tables

**Fig. 1.**
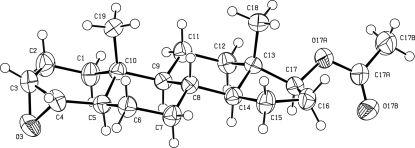
ORTEPII (Johnson, 1976) of (I). Displacement ellipsoids are drawn at the 50% probability level.

## supplementary crystallographic information

### Comment

Recently, a new series of steroids, which result from modifications in the A– and D-rings of the aromatase substrate, androstenedione, were designed, synthesized and evaluated for their anti-tumour and anti-aromatase activity (Cepa *et al.*, 2005). The researches have considered three main structural features for the drug-enzyme interactions, namely, the planarity of the A-ring, the 5α-stereochemistry and the integrity of the cyclopentanone D-ring. In the present work, we are focused on the effect of some refined modifications in the original C–17 carbonyl group in the D-ring of steroids in enzyme inhibition. This study is a contribution to the understanding of the role of the D-ring substitution pattern and its structure in the inhibition of aromatase. Under this project, the title compound (I) was synthesized. In order to establish the conformation of (I), the X-ray structure was determined (Fig. 1). Atomic distances are within expected values (Allen *et al.*, 1987) except for the C2–C3 bond which is much shorter [1.482 (5) Å] than the determined average value for C*sp*^3^–C*sp*^3^ bond lengths in the molecule [1.532 (16) Å]. This is probably characteristic of 3–4 epoxides since similar values [1.494 (4) and 1.484 (5) Å] were obtained for two related structures (Paixão *et al.*, 1997 and Andrade *et al.*, 1997). The ring A [C1→C10] is severely distorted assuming a conformation intermediate between10β-sofa and 1α,10β-half chair [asymmetry parametres (Duax & Norton, 1975): ΔC_s_(3)=11.4 (3), ΔC_2_(3,4)=14.8 (4) and ΔC_2_(1,2)=53.5 (4)°]. Rings B [C5→C10] and C [C8→-C14] have slightly flattened chair conformations evidenced by the average values of their torsion angles [56 (2)° for both]. The five member ring D [C13→C17] assumes a conformation intermediate between 13β-envelope and 13β,14α-half chair, probably due to the acetoxy substituent [puckering parametres (Cremer & Pople, 1975) q_2_=0.477 (3)Å and φ_2_=188.6 (4)°; pseudo-rotation (Altona *et al.*, 1968) and asymmetry parameters (Duax & Norton, 1975): Δ=18.8 (4), φ_m_=48.5 (2), ΔC_s_(13)=8.9 (3), ΔC_2_(13,14)=12.7 (3) and ΔC_s_(14)=27.2 (3)°). The distance between terminal O atoms is 11.204 (3)°. A pseudo-torsion angle C19–C10···C13–C18 of 1.6 (2) ° evidences that the molecule is not twisted. The dihedral angle between the least-squares plane of the four non-H atoms of the acetate group and that of ring D is 65.04 (17)°. The crystal packing is determined by van der Waals interactions.

### Experimental

To a solution of 5α-androst-3-en-17β-yl acetate (308 mg, 0.97 mmol) in methylene chloride (5.0 ml), a solution of performic acid (0.15 ml of HCOOH 98–100% and 0.4 ml of H_2_O_2_ 35%) was added and the reaction stirred overnight until complete transformation of starting material. Methylene chloride (150 ml) was added and the organic layer was washed with 10% NaHCO_3_ (2x100 ml) and water (4x100 ml) and then dried over anhydrous MgSO_4_. After filtration and solvent evaporation to dryness, the almost pure title compound was obtained as a white solid (296 mg, 92%). Column chromatography (silica gel 60 with 95: 5 to 95: 10 mixtures of petroleum ether 40–60 °C and ethyl acetate) or crystallization from ethyl acetate *n*–hexane, yielded analytical samples: Mp 461–463 K; IR ν_max_(KBr) cm^-1^: 1732 (C?O); ^1^H NMR (300 MHz, CDCl_3_) δ: 0.77 (3*H*, s, 18-H_3_)*, 0.78 (3*H*, s, 19-H_3_)*, 2.03 (3*H*, s, CH_3_COO), 2.69 (1*H*, d, *J*_4__β__,5__α_=3.9, 4β-H), 3.16 (1*H*, dd, *J*_3__β__,2__α_=3.0, *J*_3__β__,2__β_=3.0, 3β-H), 4.58 (1*H*, dd, *J*_17__α__,16__α_=9.0, *J*_17__α__,16__β_=7.8, 17α-H); ^13^C NMR (75.6 MHz, DMSO-d_6_) δ: 12.1 (C-19), 13.4 (C-18), 20.7, 21.2, 21.3, 23.4, 26.6, 27.5, 30.4, 31.4, 34.1, 35.1, 36.8, 42.6, 46.7, 50.5 52.1**, 52.5 (C-4)**, 55.8 (C-3), 82.7 (C-17); 171.2 (C?O); EIMS *m/z* 332 (*M*^+^, 87%). *,** Signals may be interchangeable.

### Refinement

All hydrogen atoms were refined as riding on their parent atoms using *SHELXL97*. The absolute configuration was not determined from the X-ray data but was known from the synthesis route. Friedel pairs were merged before refinement.

### Crystal data

**Table:**

C_21_H_32_O_3_	*D*_x_ = 1.196 Mg m^−^^3^
*M**_r_* = 332.47	Cu *K*α radiation, λ = 1.54180 Å
Orthorhombic, *P*2_1_2_1_2_1_	Cell parameters from 25 reflections
*a* = 6.2760 (2) Å	θ = 13.4–28.2°
*b* = 11.7272 (19) Å	µ = 0.61 mm^−^^1^
*c* = 25.0888 (9) Å	*T* = 293 K
*V* = 1846.5 (3) Å^3^	Truncated pyramid, colourless
*Z* = 4	0.36 × 0.20 × 0.12 mm
*F*(000) = 728	

### Data collection

**Table:**

Enraf–Nonius MACH-3 diffractometer	*R*_int_ = 0.050
Radiation source: fine-focus sealed tube	θ_max_ = 73.8°, θ_min_ = 3.5°
graphite	*h* = −6→7
Profile data from ω–2θ scans	*k* = 0→14
2661 measured reflections	*l* = 0→31
2146 independent reflections	3 standard reflections every 300 reflections
1715 reflections with *I* > 2σ(*I*)	intensity decay: 1.3%

### Refinement

**Table:**

Refinement on *F*^2^	Hydrogen site location: inferred from neighbouring sites
Least-squares matrix: full	H-atom parameters constrained
*R*[*F*^2^ > 2σ(*F*^2^)] = 0.044	*w* = 1/[σ^2^(*F*_o_^2^) + (0.0886*P*)^2^ + 0.2976*P*] where *P* = (*F*_o_^2^ + 2*F*_c_^2^)/3
*wR*(*F*^2^) = 0.135	(Δ/σ)_max_ = 0.004
*S* = 1.03	Δρ_max_ = 0.23 e Å^−^^3^
2146 reflections	Δρ_min_ = −0.18 e Å^−^^3^
221 parameters	Extinction correction: *SHELXL97* (Sheldrick, 2008), Fc^*^=kFc[1+0.001xFc^2^λ^3^/sin(2θ)]^-1/4^
0 restraints	Extinction coefficient: 0.0030 (6)
Primary atom site location: structure-invariant direct methods	Absolute structure: Flack (1983), 0 Friedel pairs
Secondary atom site location: difference Fourier map	Flack parameter: −0.1 (5)

### Special details

**Table:**

Geometry. All e.s.d.'s (except the e.s.d. in the dihedral angle between two l.s. planes) are estimated using the full covariance matrix. The cell e.s.d.'s are taken into account individually in the estimation of e.s.d.'s in distances, angles and torsion angles; correlations between e.s.d.'s in cell parameters are only used when they are defined by crystal symmetry. An approximate (isotropic) treatment of cell e.s.d.'s is used for estimating e.s.d.'s involving l.s. planes.
Refinement. Refinement of *F*^2^ against ALL reflections. The weighted *R*-factor *wR* and goodness of fit *S* are based on *F*^2^, conventional *R*-factors *R* are based on *F*, with *F* set to zero for negative *F*^2^. The threshold expression of *F*^2^ > σ(*F*^2^) is used only for calculating *R*-factors(gt) *etc*. and is not relevant to the choice of reflections for refinement. *R*-factors based on *F*^2^ are statistically about twice as large as those based on *F*, and *R*- factors based on ALL data will be even larger.

### Fractional atomic coordinates and isotropic or equivalent isotropic displacement parameters (Å^2^)

**Table:**

	*x*	*y*	*z*	*U*_iso_*/*U*_eq_	
O3	0.6940 (5)	0.54519 (19)	0.50056 (8)	0.0627 (7)	
O17A	1.1217 (4)	0.38347 (16)	0.12529 (7)	0.0536 (6)	
O17B	1.2588 (5)	0.52535 (19)	0.07703 (9)	0.0669 (7)	
C1	0.9480 (5)	0.3851 (3)	0.42667 (10)	0.0481 (7)	
H1A	1.0406	0.3237	0.4150	0.058*	
H1B	1.0340	0.4535	0.4298	0.058*	
C2	0.8561 (6)	0.3552 (3)	0.48152 (11)	0.0565 (8)	
H2A	0.8167	0.2752	0.4816	0.068*	
H2B	0.9667	0.3655	0.5081	0.068*	
C3	0.6677 (6)	0.4235 (3)	0.49734 (11)	0.0529 (8)	
H3	0.5753	0.3892	0.5244	0.063*	
C4	0.5617 (5)	0.4987 (3)	0.45865 (10)	0.0480 (7)	
H4	0.4075	0.5083	0.4630	0.058*	
C5	0.6477 (5)	0.5113 (2)	0.40261 (9)	0.0381 (6)	
H5	0.7500	0.5744	0.4038	0.046*	
C6	0.4757 (5)	0.5465 (2)	0.36317 (10)	0.0436 (6)	
H6A	0.4018	0.6134	0.3763	0.052*	
H6B	0.3726	0.4854	0.3594	0.052*	
C7	0.5761 (5)	0.5727 (2)	0.30914 (10)	0.0431 (6)	
H7A	0.6666	0.6393	0.3124	0.052*	
H7B	0.4643	0.5904	0.2837	0.052*	
C8	0.7080 (4)	0.4730 (2)	0.28806 (9)	0.0338 (5)	
H8	0.6114	0.4090	0.2812	0.041*	
C9	0.8761 (4)	0.4339 (2)	0.32915 (9)	0.0340 (5)	
H9	0.9703	0.4993	0.3352	0.041*	
C10	0.7745 (4)	0.4048 (2)	0.38411 (9)	0.0352 (5)	
C11	1.0165 (4)	0.3385 (2)	0.30642 (10)	0.0407 (6)	
H11A	0.9306	0.2704	0.3018	0.049*	
H11B	1.1282	0.3209	0.3318	0.049*	
C12	1.1185 (5)	0.3702 (3)	0.25284 (10)	0.0429 (6)	
H12A	1.2172	0.4329	0.2581	0.051*	
H12B	1.1983	0.3056	0.2393	0.051*	
C13	0.9502 (4)	0.4047 (2)	0.21221 (9)	0.0355 (6)	
C14	0.8196 (4)	0.5035 (2)	0.23599 (9)	0.0359 (6)	
H14	0.9218	0.5639	0.2446	0.043*	
C15	0.6882 (5)	0.5471 (3)	0.18884 (10)	0.0501 (7)	
H15A	0.6508	0.6267	0.1935	0.060*	
H15B	0.5587	0.5028	0.1846	0.060*	
C16	0.8385 (5)	0.5311 (3)	0.14039 (10)	0.0496 (7)	
H16A	0.7686	0.4879	0.1125	0.060*	
H16B	0.8819	0.6044	0.1261	0.060*	
C17	1.0306 (5)	0.4658 (2)	0.16230 (10)	0.0427 (6)	
H17	1.1411	0.5206	0.1726	0.051*	
C17A	1.2337 (5)	0.4256 (3)	0.08422 (11)	0.0496 (7)	
C17B	1.3214 (8)	0.3334 (3)	0.05006 (14)	0.0751 (12)	
H17A	1.4147	0.3659	0.0238	0.090*	
H17B	1.2066	0.2943	0.0326	0.090*	
H17C	1.3996	0.2805	0.0717	0.090*	
C18	0.8135 (6)	0.3021 (2)	0.19638 (12)	0.0507 (7)	
H18A	0.9021	0.2450	0.1802	0.061*	
H18B	0.7062	0.3258	0.1715	0.061*	
H18C	0.7464	0.2710	0.2275	0.061*	
C19	0.6299 (5)	0.2992 (2)	0.38040 (11)	0.0433 (6)	
H19A	0.7163	0.2318	0.3787	0.052*	
H19B	0.5435	0.3043	0.3489	0.052*	
H19C	0.5398	0.2957	0.4113	0.052*	

### Atomic displacement parameters (Å^2^)

**Table:**

	*U*^11^	*U*^22^	*U*^33^	*U*^12^	*U*^13^	*U*^23^
O3	0.0852 (18)	0.0598 (13)	0.0431 (10)	−0.0031 (14)	−0.0105 (12)	−0.0122 (10)
O17A	0.0745 (15)	0.0438 (10)	0.0426 (10)	0.0005 (11)	0.0153 (11)	−0.0037 (9)
O17B	0.0881 (18)	0.0506 (12)	0.0620 (13)	−0.0109 (14)	0.0256 (14)	0.0019 (11)
C1	0.0472 (15)	0.0599 (17)	0.0371 (13)	0.0056 (15)	−0.0072 (13)	0.0053 (13)
C2	0.066 (2)	0.0656 (19)	0.0380 (13)	0.0059 (18)	−0.0084 (15)	0.0076 (13)
C3	0.066 (2)	0.0574 (17)	0.0348 (13)	−0.0033 (17)	0.0006 (14)	−0.0005 (12)
C4	0.0508 (16)	0.0553 (16)	0.0378 (13)	0.0003 (15)	0.0003 (13)	−0.0072 (12)
C5	0.0395 (14)	0.0380 (13)	0.0368 (12)	−0.0002 (12)	−0.0013 (11)	0.0000 (10)
C6	0.0413 (14)	0.0473 (14)	0.0421 (13)	0.0122 (13)	0.0042 (12)	0.0019 (11)
C7	0.0446 (15)	0.0449 (14)	0.0397 (13)	0.0113 (13)	0.0003 (12)	0.0080 (11)
C8	0.0317 (11)	0.0353 (12)	0.0343 (11)	0.0004 (11)	−0.0021 (10)	0.0025 (9)
C9	0.0315 (11)	0.0355 (12)	0.0349 (11)	−0.0004 (11)	−0.0032 (10)	0.0012 (10)
C10	0.0368 (13)	0.0347 (12)	0.0340 (11)	0.0015 (12)	−0.0027 (11)	0.0032 (9)
C11	0.0394 (13)	0.0445 (14)	0.0381 (12)	0.0095 (13)	−0.0041 (11)	0.0034 (11)
C12	0.0380 (14)	0.0496 (15)	0.0411 (13)	0.0068 (13)	0.0014 (12)	−0.0026 (11)
C13	0.0383 (13)	0.0330 (12)	0.0351 (12)	−0.0018 (11)	−0.0009 (11)	−0.0022 (10)
C14	0.0362 (13)	0.0359 (12)	0.0357 (12)	0.0005 (11)	−0.0028 (11)	0.0024 (10)
C15	0.0516 (16)	0.0585 (17)	0.0401 (13)	0.0086 (15)	−0.0038 (14)	0.0099 (13)
C16	0.0633 (19)	0.0504 (15)	0.0351 (12)	0.0043 (16)	−0.0023 (13)	0.0052 (12)
C17	0.0508 (15)	0.0402 (13)	0.0372 (12)	−0.0050 (14)	0.0065 (12)	−0.0037 (11)
C17A	0.0558 (18)	0.0517 (16)	0.0414 (13)	−0.0010 (15)	0.0088 (14)	−0.0001 (12)
C17B	0.110 (3)	0.056 (2)	0.0590 (19)	0.011 (2)	0.030 (2)	−0.0007 (16)
C18	0.0618 (19)	0.0430 (14)	0.0474 (15)	−0.0150 (15)	−0.0002 (15)	−0.0022 (12)
C19	0.0489 (15)	0.0384 (13)	0.0427 (13)	−0.0039 (13)	0.0024 (13)	0.0039 (11)

### Geometric parameters (Å, °)

**Table:**

O3—C3	1.439 (4)	C9—H9	0.9800
O3—C4	1.447 (3)	C10—C19	1.537 (4)
O17A—C17A	1.342 (3)	C11—C12	1.535 (4)
O17A—C17	1.457 (3)	C11—H11A	0.9700
O17B—C17A	1.194 (4)	C11—H11B	0.9700
C1—C2	1.533 (4)	C12—C13	1.523 (4)
C1—C10	1.543 (4)	C12—H12A	0.9700
C1—H1A	0.9700	C12—H12B	0.9700
C1—H1B	0.9700	C13—C17	1.528 (3)
C2—C3	1.482 (5)	C13—C18	1.531 (4)
C2—H2A	0.9700	C13—C14	1.539 (4)
C2—H2B	0.9700	C14—C15	1.530 (3)
C3—C4	1.471 (4)	C14—H14	0.9800
C3—H3	0.9800	C15—C16	1.550 (4)
C4—C5	1.513 (4)	C15—H15A	0.9700
C4—H4	0.9800	C15—H15B	0.9700
C5—C6	1.521 (4)	C16—C17	1.530 (4)
C5—C10	1.553 (4)	C16—H16A	0.9700
C5—H5	0.9800	C16—H16B	0.9700
C6—C7	1.526 (4)	C17—H17	0.9800
C6—H6A	0.9700	C17A—C17B	1.485 (4)
C6—H6B	0.9700	C17B—H17A	0.9600
C7—C8	1.526 (4)	C17B—H17B	0.9600
C7—H7A	0.9700	C17B—H17C	0.9600
C7—H7B	0.9700	C18—H18A	0.9600
C8—C14	1.525 (3)	C18—H18B	0.9600
C8—C9	1.545 (3)	C18—H18C	0.9600
C8—H8	0.9800	C19—H19A	0.9600
C9—C11	1.534 (4)	C19—H19B	0.9600
C9—C10	1.557 (3)	C19—H19C	0.9600
			
C3—O3—C4	61.3 (2)	C9—C11—H11A	109.0
C17A—O17A—C17	116.8 (2)	C12—C11—H11A	109.0
C2—C1—C10	112.9 (3)	C9—C11—H11B	109.0
C2—C1—H1A	109.0	C12—C11—H11B	109.0
C10—C1—H1A	109.0	H11A—C11—H11B	107.8
C2—C1—H1B	109.0	C13—C12—C11	111.2 (2)
C10—C1—H1B	109.0	C13—C12—H12A	109.4
H1A—C1—H1B	107.8	C11—C12—H12A	109.4
C3—C2—C1	114.6 (3)	C13—C12—H12B	109.4
C3—C2—H2A	108.6	C11—C12—H12B	109.4
C1—C2—H2A	108.6	H12A—C12—H12B	108.0
C3—C2—H2B	108.6	C12—C13—C17	116.4 (2)
C1—C2—H2B	108.6	C12—C13—C18	110.7 (2)
H2A—C2—H2B	107.6	C17—C13—C18	110.0 (2)
O3—C3—C4	59.62 (19)	C12—C13—C14	108.0 (2)
O3—C3—C2	117.4 (3)	C17—C13—C14	98.1 (2)
C4—C3—C2	120.6 (3)	C18—C13—C14	113.2 (2)
O3—C3—H3	115.8	C8—C14—C15	119.5 (2)
C4—C3—H3	115.8	C8—C14—C13	113.7 (2)
C2—C3—H3	115.8	C15—C14—C13	103.8 (2)
O3—C4—C3	59.08 (18)	C8—C14—H14	106.3
O3—C4—C5	115.7 (3)	C15—C14—H14	106.3
C3—C4—C5	120.7 (3)	C13—C14—H14	106.3
O3—C4—H4	116.3	C14—C15—C16	103.8 (2)
C3—C4—H4	116.3	C14—C15—H15A	111.0
C5—C4—H4	116.3	C16—C15—H15A	111.0
C4—C5—C6	112.2 (2)	C14—C15—H15B	111.0
C4—C5—C10	112.4 (2)	C16—C15—H15B	111.0
C6—C5—C10	112.8 (2)	H15A—C15—H15B	109.0
C4—C5—H5	106.3	C17—C16—C15	105.0 (2)
C6—C5—H5	106.3	C17—C16—H16A	110.8
C10—C5—H5	106.3	C15—C16—H16A	110.8
C5—C6—C7	109.8 (2)	C17—C16—H16B	110.8
C5—C6—H6A	109.7	C15—C16—H16B	110.8
C7—C6—H6A	109.7	H16A—C16—H16B	108.8
C5—C6—H6B	109.7	O17A—C17—C13	109.9 (2)
C7—C6—H6B	109.7	O17A—C17—C16	114.3 (2)
H6A—C6—H6B	108.2	C13—C17—C16	105.6 (2)
C6—C7—C8	112.2 (2)	O17A—C17—H17	109.0
C6—C7—H7A	109.2	C13—C17—H17	109.0
C8—C7—H7A	109.2	C16—C17—H17	109.0
C6—C7—H7B	109.2	O17B—C17A—O17A	123.1 (3)
C8—C7—H7B	109.2	O17B—C17A—C17B	125.2 (3)
H7A—C7—H7B	107.9	O17A—C17A—C17B	111.7 (3)
C14—C8—C7	111.5 (2)	C17A—C17B—H17A	109.5
C14—C8—C9	109.1 (2)	C17A—C17B—H17B	109.5
C7—C8—C9	111.5 (2)	H17A—C17B—H17B	109.5
C14—C8—H8	108.2	C17A—C17B—H17C	109.5
C7—C8—H8	108.2	H17A—C17B—H17C	109.5
C9—C8—H8	108.2	H17B—C17B—H17C	109.5
C11—C9—C8	111.17 (19)	C13—C18—H18A	109.5
C11—C9—C10	113.9 (2)	C13—C18—H18B	109.5
C8—C9—C10	112.1 (2)	H18A—C18—H18B	109.5
C11—C9—H9	106.4	C13—C18—H18C	109.5
C8—C9—H9	106.4	H18A—C18—H18C	109.5
C10—C9—H9	106.4	H18B—C18—H18C	109.5
C19—C10—C1	109.8 (2)	C10—C19—H19A	109.5
C19—C10—C5	111.3 (2)	C10—C19—H19B	109.5
C1—C10—C5	106.0 (2)	H19A—C19—H19B	109.5
C19—C10—C9	111.4 (2)	C10—C19—H19C	109.5
C1—C10—C9	110.9 (2)	H19A—C19—H19C	109.5
C5—C10—C9	107.36 (19)	H19B—C19—H19C	109.5
C9—C11—C12	112.8 (2)		
			
C10—C1—C2—C3	−42.1 (4)	C11—C9—C10—C5	177.3 (2)
C4—O3—C3—C2	111.1 (3)	C8—C9—C10—C5	−55.4 (3)
C1—C2—C3—O3	−59.3 (4)	C8—C9—C11—C12	53.4 (3)
C1—C2—C3—C4	9.8 (4)	C10—C9—C11—C12	−178.8 (2)
C3—O3—C4—C5	−111.8 (3)	C9—C11—C12—C13	−55.7 (3)
C2—C3—C4—O3	−105.8 (4)	C11—C12—C13—C17	165.4 (2)
O3—C3—C4—C5	103.4 (3)	C11—C12—C13—C18	−68.1 (3)
C2—C3—C4—C5	−2.4 (5)	C11—C12—C13—C14	56.4 (3)
O3—C4—C5—C6	−137.4 (3)	C7—C8—C14—C15	−55.3 (3)
C3—C4—C5—C6	154.8 (3)	C9—C8—C14—C15	−178.9 (2)
O3—C4—C5—C10	94.2 (3)	C7—C8—C14—C13	−178.4 (2)
C3—C4—C5—C10	26.4 (4)	C9—C8—C14—C13	57.9 (3)
C4—C5—C6—C7	172.8 (2)	C12—C13—C14—C8	−59.6 (3)
C10—C5—C6—C7	−59.0 (3)	C17—C13—C14—C8	179.2 (2)
C5—C6—C7—C8	55.4 (3)	C18—C13—C14—C8	63.4 (3)
C6—C7—C8—C14	−176.0 (2)	C12—C13—C14—C15	169.0 (2)
C6—C7—C8—C9	−53.8 (3)	C17—C13—C14—C15	47.8 (2)
C14—C8—C9—C11	−53.0 (3)	C18—C13—C14—C15	−68.0 (3)
C7—C8—C9—C11	−176.7 (2)	C8—C14—C15—C16	−162.5 (2)
C14—C8—C9—C10	178.26 (19)	C13—C14—C15—C16	−34.7 (3)
C7—C8—C9—C10	54.6 (3)	C14—C15—C16—C17	7.2 (3)
C2—C1—C10—C19	−56.1 (3)	C17A—O17A—C17—C13	−168.1 (2)
C2—C1—C10—C5	64.3 (3)	C17A—O17A—C17—C16	73.4 (3)
C2—C1—C10—C9	−179.5 (2)	C12—C13—C17—O17A	78.1 (3)
C4—C5—C10—C19	64.4 (3)	C18—C13—C17—O17A	−48.7 (3)
C6—C5—C10—C19	−63.7 (3)	C14—C13—C17—O17A	−167.0 (2)
C4—C5—C10—C1	−54.9 (3)	C12—C13—C17—C16	−158.1 (2)
C6—C5—C10—C1	177.0 (2)	C18—C13—C17—C16	75.1 (3)
C4—C5—C10—C9	−173.4 (2)	C14—C13—C17—C16	−43.3 (2)
C6—C5—C10—C9	58.5 (3)	C15—C16—C17—O17A	144.0 (3)
C11—C9—C10—C19	−60.6 (3)	C15—C16—C17—C13	23.0 (3)
C8—C9—C10—C19	66.7 (3)	C17—O17A—C17A—O17B	−0.7 (5)
C11—C9—C10—C1	62.0 (3)	C17—O17A—C17A—C17B	178.9 (3)
C8—C9—C10—C1	−170.7 (2)	C19—C10—C13—C18	1.6 (2)
